# Munchausen syndrome revealed by subcutaneous limb emphysema: a case report

**DOI:** 10.1186/s13256-015-0649-x

**Published:** 2015-08-18

**Authors:** Kaldadak Koufagued, Bouchaib Chafry, Youssef Benyass, Yves Abissegue, Driss Benchebba, Salim Bouabid, Chagar Belkacem

**Affiliations:** Department of Orthopedic Trauma, Mohamed V Military Hospital, University Mohamed V- souissi Rabat Morocco, S/C ERSSM BP 1044, Rabat Océan, Morocco

**Keywords:** Subcutaneous emphysema, Limb, Factitious disorder, Munchausen syndrome

## Abstract

**Introduction:**

Limb subcutaneous emphysema secondary to a Munchausen syndrome represents a rare and severe entity because it involves the functional prognosis of the limb and vital prognosis of the patient.

**Case presentation:**

We report the case of an 18-year-old Moroccan woman patient who presented to our hospital with a subcutaneous emphysema of the shoulder girdle and the right arm, caused by our patient. Treatment was aggressive, with a wide surgical debridement, parenteral antibiotic therapy and hyperbaric oxygen therapy. The results have been favorable.

**Conclusions:**

The correlation of anamnestic data and clinical and para-clinical exams were essential for the diagnosis of Munchausen syndrome in this case. In this regard, we report a rare case of subcutaneous limb emphysema secondary to Munchausen syndrome.

## Introduction

Munchausen syndrome is a factitious disorder that involves falsification of psychological or physical signs or symptoms caused entirely by the patient themselves, in a clear state of consciousness, in order to play the role of a sick person. Factitious disorders are rare: less than 1% of patients [1]. The criteria for the diagnosis of factitious disorders are found in the Diagnostic and Statistical Manual of Mental Disorders Fourth Edition, Text Revision (DSM-IV TR) [2]. Isolated subcutaneous limb emphysema secondary to Munchausen syndrome is a rare entity [3, 4], and it may have adverse consequences on the limb and patient prognosis, leading to aggressive treatments. We report a case of Munchausen syndrome revealed by subcutaneous emphysema of the shoulder region and the right arm, which required an aggressive management.

## Case report

**Fig. 1:**
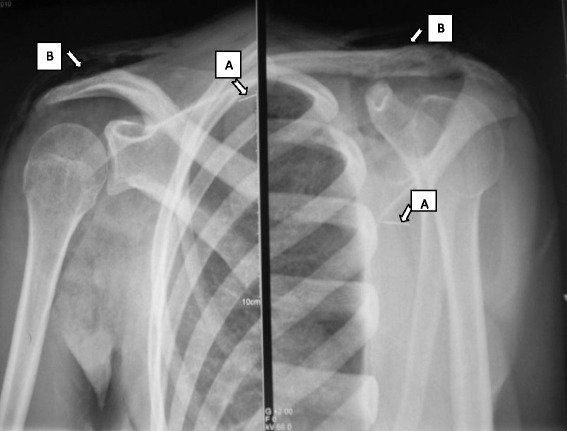
Front and three quarters shoulder radiography showing the presence of the needle in the subscapularis (arrow A) and subcutaneous emphysema in the shoulder (arrow B)

**Fig. 2:**
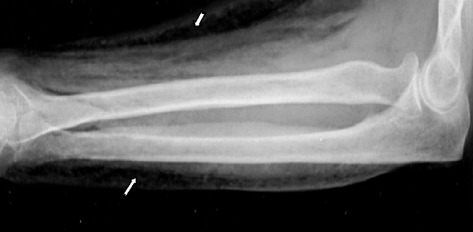
X-ray profile of her right arm showing subcutaneous emphysema (arrows)

An 18-year-old Moroccan woman with no particular medical history presented to our hospital with a painful swelling of the right arm. Her examination found no signs of trauma and the blood pressure, heart and respiratory rate were normals. Her physical examination revealed a swollen and painful shoulder with warm shoulder pain. On palpation there was subcutaneous crepitation spreading to her arm, forearm and chest; the distal pulse was present, but there was no redness or necrotic areas. The joints of her shoulder and elbow were free and in a context of apyrexia, and in a well-preserved general condition. Her laboratory tests showed leukocytosis at 11,000 elements per mm^3^. Her sedimentation rate and C-reactive protein (CRP) level were normal. Plain radiographs (Figs. 1 and 2) of her shoulder showed the presence of a foreign body and subcutaneous gas in the soft tissues of the right scapular region, spreading to her arm and forearm.

**Fig. 3:**
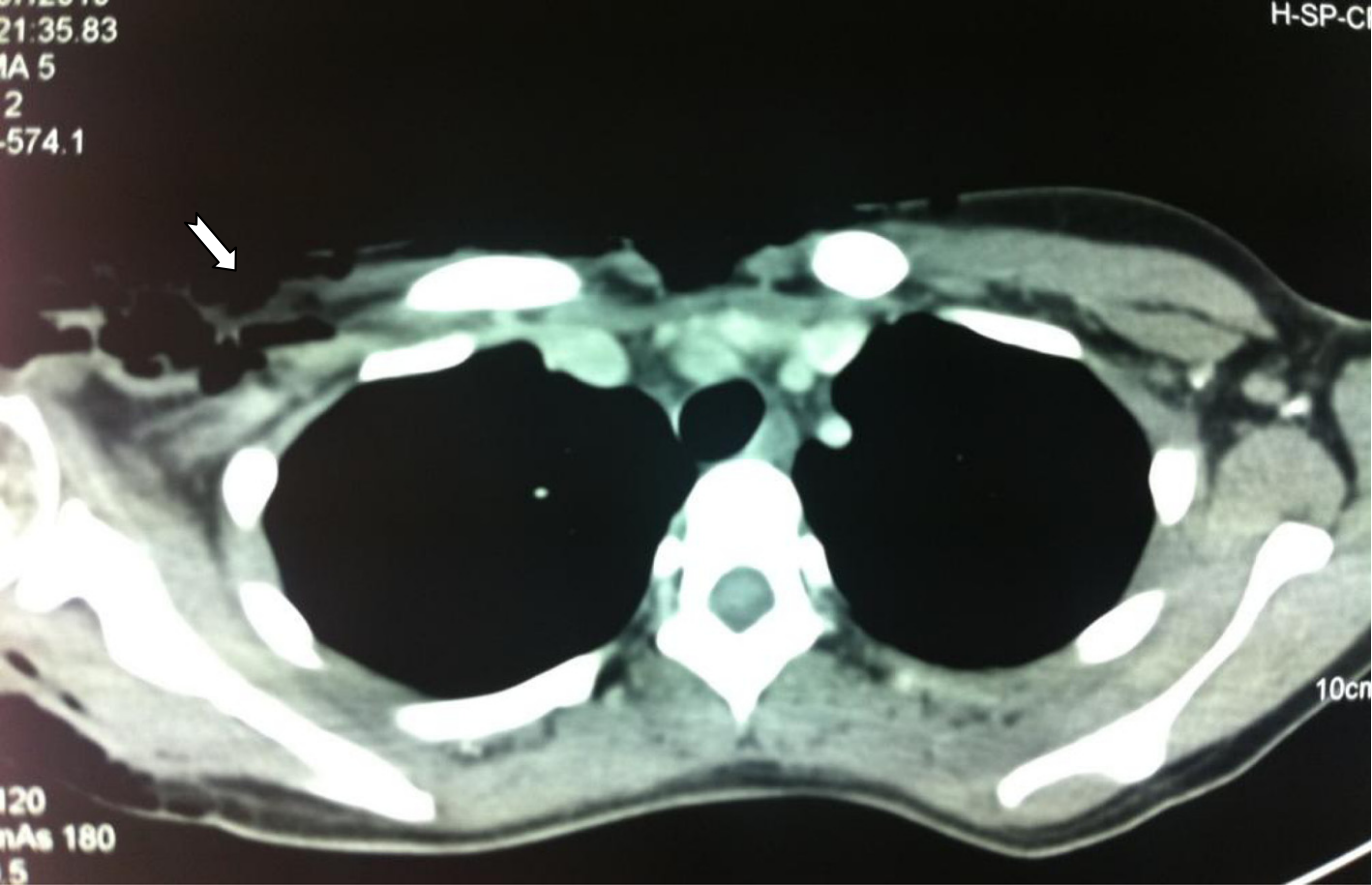
Thoracic computed tomography image showing a diffuse subcutaneous emphysema (arrow showing emphysema)

Computed tomography (CT) images (Fig. 3) showed the presence of a right retro scapular para-median needle, measuring 10mm in length, and a diffuse parietal emphysema of her shoulder and right arm and her anterior chest wall and trunk, and beneath her right breast. Given the strong possibility of the diagnosis of gas gangrene, surgical debridement was performed.

Surgical exploration found no foul odor in the subcutaneous tissue (which was normal) and muscle fascia. The opening of the exertional compartment showed slightly discolored stuffed muscles and gas bubbles, but without pus or necrotic areas. Bacteriological samples were taken and sent to the bacteriology laboratory for examination and tests. A course of triple-combination broad spectrum antibiotics was administered for 10 days. The results of the bacteriological sampling were sterile. She received post-operative sessions of hyperbaric oxygen therapy at the rate of one session of 45 minutes per day for 10 days, and daily care in the operating room. Her post-operative follow-up was very satisfactory, with resolution of her leukocytosis.

The quarrelsome and aggressive nature of our patient, her difficult relationship with the caregiver, records of her consultation for the same symptoms in four other hospitals, a lack of biological and clinical signs, and her inability to provide an explanation for the presence of the needle and the swelling in her right arm made us suspicions about the possibility of a factitious disease. Rigorous questioning had pushed our patient to admit that she had voluntarily introduced the needle, explaining the presence of a foreign body in the right shoulder.

A psychiatric consultation was requested to confirm the diagnosis of a factitious disorder and treatment. Maltreatment history and emotional abuse during childhood were reported. Her psychiatric report stipulated that the act of self-injection of the needle and her need to draw attention to this was largely involuntary, whereas her trickery and simulation of a disease were involuntary and with no external gain or incentive therefore confirming munchausen syndrome.

## Discussion

Gas gangrene is an infectious disease constituting a therapeutic emergency; it may affect the functional prognosis of the affected limb or even the life of the patient [5]. We illustrate how, despite the importance of the presence of gas in a limb, the correlation between a patient's history and clinical and laboratory observations are crucial to avoid unnecessary aggressive treatment. Isolated subcutaneous limb emphysema is rare [3, 4], and in most cases it is secondary to traumatic or infectious disease. However, there are cases where it may appear as a result of gastrointestinal, genitourinary or lung lacerations [6].

The diagnosis of factitious illness is certainly not well known by medical personnel, and its evocation is not routinely evoked. A study [7] reported that in patients with white blood cell count, less than 15.4×10^9^ per liter and natremia less than 135mmol/L; the risk of developing a factitious disorder was 1%. This is particularly useful in cases similar to ours where the symptoms were minimal and biological parameters were within normal.

The DSM-IV-TR requires that the following three criteria be met for the diagnosis of factitious disorder: (1) intentional production or feigning of physical or psychological signs or symptoms, (2) motivation for the behavior is to assume the sick role, and (3) absence of external incentives for the behavior (such as economic gain, avoiding legal responsibility, or improving physical well-being, as in malingering) [2].

In the absence of trauma, local or general signs and laboratory abnormalities, the possibility of self-subcutaneous air injection by the patient themselves should be considered (by Munchausen syndrome in this case), especially nowadays when the availability of needles and syringes to the general population is becoming greater. The presence of puncture marks, very localized circular pockets and a bilateral or recurrent presentation should arouse strong suspicions about the possibility of a fictitious disease [8]. In our patient, no bite mark was visible on clinical examination and we noted the presence of a foreign body in the right shoulder by imaging.

It is important to stress the difference between faking a pathology and simulation. In factitious disorders, the patient deliberately acts to produce symptoms in order to assume the sick role. The actions are not consciously deliberate and the patient does not aspire to external gains [9]. This contrasts with the simulation, in which external gains are the primary motivation for the induction of the disease itself.

Our patient had a history of maltreatment, abuse and emotional abuse from childhood. Her motivation for deliberate self-injection needle and attention seeking were not pursuing external gains and were largely unconscious. She therefore fulfilled the criteria for diagnosis of Munchausen syndrome.

A good knowledge of Munchausen syndrome by health personnel will help in early identification of the DSM-IV TR criteria and a diagnosis, therefore allowing for a better treatment and prognosis. Consolidating electronic health records across hospital systems may help to identify patients earlier in the course of their illness [1].

## Conclusions

The presence of subcutaneous emphysema of a limb is disturbing and raises strong suspicions about the presence of gas gangrene, the consequences of which can be devastating. A fast and accurate diagnosis combined with appropriate, promptly initiated treatment will have a positive direct impact on the patient's limb and life prognosis. It is important to realize, as shown in our case report, that there are various causes of subcutaneous benign emphysema. An accurate and detailed history, an appropriate physical examination and a judgment that connects clinical and laboratory findings are essential and help prevent unnecessary radical treatment. If the underlying cause is not apparent, the diagnosis of factitious disorder must always be upheld.

## Consent

Written informed consent was obtained from the patient for publication of this case report and any accompanying images. A copy of the written consent is available for review by the Editor-in-Chief of this journal.

## Abbreviations

CRP: C-reactive protein
CT: Computed tomography
DSM-IV-TR: Diagnostic and Statistical Manual of Mental Disorders Fourth Edition, Text Revision)

## References

[1] Vaduganathan M, McCullough SA, Fraser TN, Stern TA. Death due to munchausen syndrome: a case of idiopathic recurrent right ventricular failure and a review of the literature. Psychosomatics. 2014;55(6):668–72. 10.1016/j.psym.2014.05.013 25262044

[2] Karadsheh MF. Bloody tears: a rare presentation of munchausen syndrome case report and review. J Family Med Prim Care. 2015;4(1):132–4. 10.4103/2249-4863.152271 PMC4366986 25811005

[3] Stevenson J. Sucking wounds of the limbs. Injury. 1995;26:151–3. 10.1016/0020-1383(95)93491-y 7744467

[4] Udell JL, Julsrud ME. Subcutaneous emphysema of the lower extremity. A case report. J Am Podiatr Med Assoc. 1990;80:446–7. 10.7547/87507315-80-8-446 2376837

[5] Headley AJ. Necrotizing soft tissue infections: a primary care review. Am Fam Physician. 2003;68:323–8. 12892352

[6] Rauh JL. Letter: Self-induced subcutaneous emphysema in an adolescent. J Pediatr. 1976;88(4):690–1. 10.1016/s0022-3476(76)80039-x 1255332

[7] Wall DB, Klein SR, Black S, de Virgilio C. A simple model to help distinguish necrotizing fasciitis from nonnecrotizing soft tissue infection. J Am Coll Surg. 2000;191:227–31. 10.1016/s1072-7515(00)00318-5 10989895

[8] Samlaska CP, Maggio KL. Subcutaneous emphysema. Adv Dermatol. 1996;11:117–51. 8718476

[9] Mahir M, Chloros GD, Feridun C, Semiz UB, Kiral A. Emphysème sous-cutané factice d’un membre. Rev Rhum. 2008;75(1):87–90.

